# Oral manifestations in hospital-admitted COVID-19 patients: a case control study

**DOI:** 10.3389/froh.2023.1180017

**Published:** 2023-08-10

**Authors:** Sana Alhamed, Suad Aljohani

**Affiliations:** Department of Oral Diagnostic Sciences, Faculty of Dentistry, King Abdulaziz University, Jeddah, Saudi Arabia

**Keywords:** COVID-19, oral manifestations, oral signs and symptoms, oral ulcerations, taste alteration, candidal infection

## Abstract

The oral cavity can present early manifestations of several systemic diseases. Since the emergence of the COVID-19 pandemic, there have been many published studies reporting the direct effect of the virus on orofacial structures. In the present study, oral signs and symptoms of 22 hospital-admitted COVID-19 patients were examined and compared to a matching control group. Loss of taste and smell was the most prevalent symptom (65%), followed by oral dryness (45%) and halitosis (30%). The most common oral lesions were candidal infections (68%). Other less common manifestations were oral ulcerations (36%) followed by the appearance of white patches (27.3%). There was a statistically significant association between candidal infection and age in the study group, where the *p*-value was 0.008. In the present study, 80% of those who had candida infections were aged 60 years or above. There was no significant association with comorbidities such as diabetes mellitus and hypertension.

## Introduction

Severe acute respiratory syndrome coronavirus 2 [SARS-CoV-2], known as the COVID-19 pandemic, is a highly contagious infection caused by a novel single-chain RNA virus with an incubation period of 1–14 days (1–4). The disease can vary from mild flu-like to severe respiratory distress, with the first symptoms, namely, weakness, fever, headache, sore throat, cough, runny nose, abdominal pain, vomiting, and diarrhea, manifesting within 4–5 days (5). Dermatological findings were reported in the form of petechia-like and vesiculobullous lesions, erythematous lesions, and urticarial rash (2, 6). Orofacial manifestations include olfactory dysfunction and anosmia as initial symptoms (7). Oral epithelial cells offer viral entry via the angiotensin-converting enzyme 2 receptor, which facilitate viral replication and can result in tissue inflammation (4). A variable spectrum of oral mucosal manifestations has been reported in the literature, including ulcers, erosions, vesicles, pustules, fissured or depapillated tongue, maculopapular lesions, plaque (8–13), pigmentation, halitosis, white lesions, hemorrhagic crusts, necrosis, erythema, and spontaneous bleeding (14–19). The most common sites of involvement were the tongue (4, 14) followed by the labial mucosa and the palate. Suggested diagnoses of the lesions were aphthous stomatitis, herpetiform lesions, candidiasis, and vasculitis.

Since the outbreak of this pandemic, there have been more than 603,711,760 confirmed cases globally, including 6,484,136 deaths according to the World Health Organization (WHO) (5). The first COVID-19-positive case in the Kingdom of Saudi Arabia was detected on 2 March 2020 (20–23). The number of cases then rapidly escalated. There were 813,986 confirmed cases as of 8 September 2022, with 801,254 recoveries. The fatality rate was 2% with 9,309 deaths (24).

Hospitalization protocols for moderate to severe cases in Saudi Arabia depend on respiratory symptoms and the presence of other comorbidities such as diabetes, cancer, immunosuppression, and obesity (25). Descriptive studies of clinical characteristics of patients with COVID-19 in Saudi Arabia demonstrated significant differences in symptoms as a direct effect of age and comorbidities (23). To date, no published data has yet investigated oral manifestations of hospital-admitted COVID-19 patients in Saudi Arabia. Oral manifestations may help guide early diagnosis of COVID-19, and the objective of this study is to identify the oral signs and symptoms associated with admitted COVID-19 patients and their prevalence in comparison to age, gender, and comorbidity-matched control group.

## Methods

### Study design

A case-control two-center study was conducted among hospitalized COVID-19 patients in King Fahad General Hospital and East Jeddah General Hospital, Jeddah, Saudi Arabia from 1 September to 30 October 2021. The ethical approval was obtained from the research and studies department, Ministry of Health, Saudi Arabia (KACST, KSA: H-02-J-002).

#### Study subjects

Inclusion criteria:

 Patients of any age admitted for COVID-19.

Exclusion criteria:

• Non-hospitalized COVID-19 patients.
• Hospitalized patients under ventilation at the time of examinations for whom oral examinations were not feasible.

The control group was selected from the dental clinics of the same hospitals and matched to the study group for age, gender, and comorbidities. Patients were interviewed and underwent intra-oral examination by two oral medicine consultants (SA and SJ) to identify any associated oral manifestations. Informed consent was obtained from each patient or his/her guardian. Patients' characteristics including age, gender, comorbidities, concomitant medications, time to onset of COVID-19, COVID-19 vaccination, and number of vaccination doses were recorded. All patients underwent two stages of screening recorded on a data collection sheet.

The patients were first asked questions about the presence and severity of the following symptoms after the onset of COVID-19:

1. Change in ability to smell or taste.
2. Orofacial pain.
3. Halitosis.
4. Oral dryness.
5. Any oral ulceration or vesicles/bullae.

The severity of symptoms was recorded using the validated visual analog scale (VAS) (26). Second, oral mucosal examinations using a disposable tongue depressor and a flashlight were performed by the two main investigators to record all possible intraoral lesions with location specifications. Scraping using gauze was done for any white lesions. Intraoral examinations were performed similarly to the control group.

### Data analysis

Statistical Package for Social Sciences computer software (SPSS for Mac, Version 28.0; IBM, 2019) was used to analyze the data. Descriptive statistics were calculated on all variables of interest to describe study variables: arithmetic means (x¯), standard deviations (SD), frequencies, and percentages. The chi-squared test (*χ*^2^) or Fisher's exact test was used to evaluate the association between two qualitative variables or to detect differences between two or more proportions where appropriate. The level of significance was *p *= 0.05. All reported *p*-values were generated from two-sided statistical tests.

## Results

### Patients

There were 22 patients included in the study: 16 (72.7%) were men and six were women (27.3%) with a mean age of 57.9 ± 16.1 years. There were 18 patients (82%) with comorbidities, 16 (88.9%) were diabetic, and 11 (61.1%) were hypertensive. The control group demonstrated the same sex distribution with matching age and comorbidities (Table 1).

**Table 1:** Patients’ characteristics.

Variables	Study group (*n*.22)		Control group (*n*.22)	
	No.	%	No.	%
Age (years)				
Less than 20 years	1	4.5	1	4.5
20 to less than 30 years	1	4.5	1	4.5
40 to less than 50 years	4	18.2	4	18.2
50 to less than 60 years	3	13.6	3	13.6
60 years and more	13	59.1	13	59.1
Mean ± SD	57.9 ± 16.1 years		57.9 ± 16.1 years	
Sex				
Male	16	72.7	16	72.7
Female	6	27.3	6	27.3
Presence of comorbidities				
No	4	18.2	4	18.2
Yes	18	81.8	18	81.8
Comorbidities				
DM	16	88.9	16	88.9
Hypertension	11	61.1	11	61.1
Immunosuppression	3	16.7	3	16.7
Asthma	1	5.6	1	5.6
Cardiac disease	1	5.6	1	5.6
Kidney transfer	1	5.6	0	100.0

**Figure 1:**
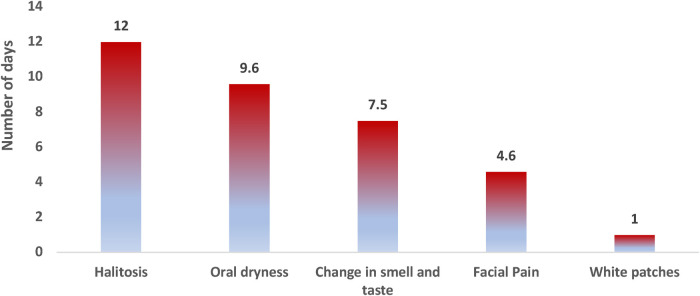
Average time in days for symptom perception to the onset of COVID-19: reported onset of halitosis was 12.0 ± 12.7 days, oral dryness was 9.6 ± 5.2 days, change of smell and taste was 7.5 ± 5.3 days, facial pain was 4.6 ± 2.0 days, and appearance of white patches was 1 ± 0.0 day after the onset of COVID-19 symptoms.

The mean period to COVID-19-confirmed diagnosis was 11.0 ± 6.3 days. In all, 20 patients (90.1%) in the study group reported one or more orofacial symptoms. Approximately two-thirds (65%) of them complained of a loss of smell and taste perception, whereas less than half (45%) presented with oral dryness (Table 2). We found that 27.3% of the study group did not receive the COVID-19 vaccine compared to 72.8% who received either one or two doses (same percentage for both). Figure 1 shows the onset of symptoms after a confirmed COVID-19 diagnosis.

**Table 2:** Distribution of the study group according to orofacial symptoms.

Presence of COVID-19 orofacial symptoms	*n*	%
No	2	9.1
Yes	20	90.9
Symptoms		
Loss of smell and taste	13	65
Oral dryness	9	45
Halitosis	6	30
Pain due to oral ulceration	2	10
Facial pain	2	10

Regarding the severity of symptoms, changes in smell and taste perception came first with a mean degree of severity of 8.9 ± 2.0 points out of 10, followed by halitosis and facial pain with the same mean score of 4.6 ± 2.3 points for both.

### Oral lesions

Approximately one-third (36.4%) of the study group had oral ulcerations while they were in the hospital. Petechiae in the palatal mucosa was presented in one patient. More than a quarter (27.3%) of the study group had white patches. More than two-thirds (68.2%) of them had candida. Table 3 details the site distribution for each lesion.

**Table 3:** Distribution of the study and control groups according to their oral manifestations.

Variables	Study group *N* (%)	Control group *N* (%)
Oral ulceration	8 (36.4)	2 (15.4)
Buccal mucosa	5 (62.5)	1 (50)
Tongue	2 (25)	1 (50)
Gingiva	1 (12.5)	0
White patches	6 (27.3)	2 (15.4)
Buccal mucosa	6 (100)	2 (100)
Lips	1 (16.7)	0
Candida infection site	15 (68.2)	4 (30.8)
Tongue	9 (60)	3 (75)
Palatal mucosa	6 (40)	1 (25)

Oral mucosal lesions were reported among more than half (59.1%) of the control group; 30.8% of these lesions were candida infection followed by tongue depapillation, white patches, ulcers, and petechiae (23.1%, 15.4%, 15.4%, and 15.4% respectively (Table 3).

Comparison between the presence of candida infection in the study group and control group denotes a statistically significant difference between them with a *p*-value of 0.001, where 68.2% of the study group had candida infection compared to only 18.2% of the control group. No statistically significant differences were noted between the two groups in terms of the presence of other oral lesions.

There was no statistically significant association between the oral lesions and comorbidities of the studied group or the control group except for ulcers and hypertension in the control group (*p *= 0.013). There was a statistically significant association between candidal infection and age in the study group, where the *p*-value was 0.008. In the present study, 80% of those who had candida infections were aged 60 years or above. There was no statistically significant association noted between candidal infection and sex and comorbidities in the study group or the control group.

There was a statistically significant association noted between the appearance of white patches and immunosuppression as one of the comorbidities of the study group (*p*-value = 0.049). However, there was no association detected among the control group.

Candidal infection was associated with the number of days to COVID-19 diagnosis, where the *p*-value was 0.006. The time to diagnosis of COVID-19 was associated with the risk of candida infection; half (53.3%) of those who had oral candidal infection were diagnosed with COVID-19 at least one week previously. However, orofacial manifestations were not associated with the COVID-19 vaccine in the study group.

## Discussion

COVID-19 infection can manifest in different body parts such as the respiratory system, gastrointestinal system, skin, and oral mucosa. In the present study, we identified the oral manifestations of hospital-admitted COVID-19 patients. The most common findings were taste disturbance and candidal infection. Other less common manifestations were oral dryness and oral ulcerations. This is in accordance with the latest update of the living systematic review conducted by dos Santos et al., as xerostomia and taste disturbance were the most common symptoms, while ulcerations were the most common oral lesions followed by herpes-like lesions, candidiasis, and angular cheilitis (27).

One of the first demonstrated symptoms of COVID-19 is taste and smell disturbance. Taste and smell disorders were found to be the most expressed symptoms in Europe but were less common in Asian patients, with an overall prevalence of 38% (27). This disturbance was found to be associated with mild/moderate COVID-19 severity. In the present study, 65% of the study group with severe COVID-19 infection complained of taste and smell disturbance with a mean severity of 8.9 ± 2. The patients reported that loss of smell and taste started 7.5 ± 5.3 days after COVID-19 onset. Giacomelli et al. reported olfactory or taste alteration in 34% of admitted COVID-19 patients with 20.3% of them having it before hospital admission and 13.5% having it during their admission (with a mean time of 6 days from COVID-19) (28).

Oral dryness is another symptom that might be related to the direct effect of the virus on salivary glands (29). Chronic medications such as oral hypoglycemic and antihypertensive medications are additional elements that can affect oral salivation. Of the study group and control group, 89% had diabetes mellitus and 61% had hypertension. Here, 45% of our study group presented with oral dryness with an onset of 9.6 days after COVID-19 diagnosis. None of the control group reported this symptom. However, there was no statistically significant association between the presence of the two comorbidities and oral dryness among the study group. This prevalence is consistent with the meta-analysis conducted by dos Santos et al., which showed a prevalence of 43% (27).

The association between oral ulceration and COVID-19 was first described by Carreras-Presas et al. (30). The impact of COVID-19 on orofacial structures may result from the direct effect of the viral infection on the oral mucosa or as a secondary result of its complications or therapeutic measures. The SARS-CoV-2 virus binding site is to the human angiotensin-converting enzyme-2 (ACE2) receptor. These are well expressed on different oral mucosal tissues such as the tongue and the palate (1, 31–33) (Xu 2020). Another theory suggests that the virus can also infect oral keratinocytes and fibroblasts, which can lead to ulceration and superficial necrosis (3). This might explain the early incidence of oral ulcerations in COVID-19 patients. Ulcerations might be directly related to anemia resulting from the direct hemolytic effect of the COVID-19 virus on red blood cells (34). In our study group, approximately one-third of the subjects had oral ulcerations while they were admitted to the hospital—this value is compatible with the findings from many other documented case reports (8, 30, 35). Only two subjects in the control group had oral ulcerations.

Candidal infection in its pseudomembranous form was the most common oral lesion in our study group (68%); only 18% of the control group showed oral candidiasis. Fungal infection in COVID-19 patients from tertiary centers in Saudi Arabia was also investigated previously (36) and showed that pseudomembranous candidiasis was the most common form of candidiasis in COVID-19 patients and was most likely related to disease comorbidities and hospital admission. COVID-19 is known to increase the risk of secondary opportunistic infections such as candidiasis. In addition, medications used to manage COVID-19, such as azithromycin, will disturb the oral microbiome and increase this risk (3). There was also a statistically significant association noted between the candida infection and the number of days to COVID-19 diagnosis and age of the study group (*p*-value = 0.006 and 0.008, respectively). It is well established that the risk of candida infection increases in elderly patients. According to the findings of the present study population**,** candida infection was found mostly in 60-year-old patients. On the other hand, no association was found between comorbidities and candidal infection as about 90% of the sample had diabetes.

There was no statistically significant association between the oral lesions and comorbidities of the studied group nor the control group except for hypertension among the control group, which can be attributed to antihypertensive medications that could result in oral mucosal lesions such as lichenoid reaction and ulcerations.

This study is limited by the small sample size. Many COVID-19 hospital-admitted patients were excluded because they were critically ill or intubated and unable to respond to the questions. Moreover, due to the nature of the study, blinding patients was not applicable. However, the results emphasize the importance of oral examinations for critically ill COVID-19 patients to diagnose and manage any related oral complaints aiming to relieve pain and improve quality of life.

## Conclusion

Candidal infection and taste disturbance were the most frequent oral lesions in hospital-admitted COVID-19 patients. Other less common manifestations were oral dryness and oral ulcerations. The exact etiopathogenesis of these manifestations is still unknown. The direct impact of the COVID-19 virus on the epithelium is the most likely cause of taste disturbance. However, other manifestations can be the result of co-infections secondary to the compromised medical status of the patients as well as the side effects of medications, such as antibiotics. Intraoral examination for hospitalized patients with COVID-19 is recommended in order to manage any related signs and symptoms.

## Data Availability

The original contributions presented in the study are included in the article/Supplementary Material, further inquiries can be directed to the corresponding author.
